# The Draft Syllabus: Official Explanation

**Published:** 1921-05-14

**Authors:** 


### The Draft Syllabus: Official Explanation.

We have received the following *précis* from the General Nursing Council, with a request for its publication:—

It is the desire of the Education and Examination Committee of the General Nursing Council for England and Wales to express their gratitude for the suggestions and criticisms at the Conference held on April 28, 1921. In most cases the criticisms of certain details of the Syllabus are being met, or have been explained and readjusted.

On page 2, paragraph 5, of the Syllabus it has been decided to recommend the Council to insert the words "District Nursing" before the words "Public Sanitation and Public Health," * and in the Syllabus for first year of training, column 3 (practical nursing), sections 3 and 8, to insert the words "preparation for" before the examinations specified [rectal and vaginal examinations].

With reference to criticisms that the first year's work is overcrowded, it should be pointed out that the subjects can be taught by a trained nurse, and that they can be woven in together in so simple a manner as to elucidate one another, and provide a general but intelligent nursing education, the details of which should be seen and taught in the wards. This arrangement seems especially suitable as a preparation for the less-educated candidate, and will be followed by the subsequent teachings of specialists and senior teachers in the second and third years of training.

In view of the position that preventive medicine is taking in the national scheme, it is surely desirable that a nurse's training should give her some knowledge of the diseases of women, and guide her as to the means of prevention and alleviation of the same, together with maternity work. It must be remembered that the majority of complications arising during and after pregnancy come under the care of a general-trained nurse.

It should be clearly understood that this Syllabus was compiled for the information of those who teach and are responsible for teaching the training nurse, and is not meant to be placed in the hands of the probationer, for whom any scientific and technical terms must necessarily be interpreted.

Public health and sanitation are subjects of such growing importance in the training of a nurse that it is deemed advisable they should be brought to her notice and kept in full view throughout her training. Hygiene and elementary science are included, as being the foundation of the nurse's work, and it is desirable to point out that in the first year of her training these subjects are taught with reference to her daily work and life in the wards.

It should be emphasised that the aim of the instruction given in the first year should be to stimulate enthusiasm, and to give the probationer an intelligent outlook, rather than to accumulate or memorise details. The material then absorbed will be enlarged during the second and third years by lectures on the same subjects and by constant experience in the wards.

It is hoped the training authorities will take up the subject of preliminary schools, the importance of which cannot be overestimated. These schools would serve their purpose as centres of training, when reciprocal training and affiliation of hospitals are recognised.

[* This addition has the effect of including district nursing, with public sanitation and public health, as one of the subjects of importance to be brought to the nurse's notice from the outset, and kept in full view throughout her training.—Ed. The Hospital.]

